# Diverse parentage relationships in paternal mouthbrooding fishes

**DOI:** 10.1098/rsbl.2021.0576

**Published:** 2022-05-04

**Authors:** Janine E. Abecia, Alison J. King, Osmar J. Luiz, David A. Crook, Dion Wedd, Sam C. Banks

**Affiliations:** ^1^ Research Institute for the Environment and Livelihoods, Charles Darwin University, Ellengowan Drive, Casuarina, NT 0810, Australia; ^2^ Centre for Freshwater Ecosystems, La Trobe University, Albury/Wodonga Campus, Vic 3690, Australia

**Keywords:** paternal, mouthbrooding, genetic parentage analysis, certainty of paternity

## Abstract

While mouthbrooding is not an uncommon parental care strategy in fishes, paternal mouthbrooding only occurs in eight fish families and is little studied. The high cost of paternal mouthbrooding to the male implies a low risk of investment in another male's offspring but genetic parentage patterns are poorly known for paternal mouthbrooders. Here, we used single-nucleotide polymorphism genetic data to investigate parentage relationships of broods of two mouthbrooders of northern Australian rivers, mouth almighty *Glossamia aprion* and blue catfish *Neoarius graeffei*. For *N. graeffei*, we found that the parentage pattern was largely monogamous with the brooder male as the sire. For *G. aprion*, the parentage pattern was more heterogeneous including observations of monogamous broods with the brooder male as the sire (73%), polygyny (13%), cuckoldry (6%) and a brood genetically unrelated to the brooder male (6%). Findings demonstrate the potential for complex interrelationships of male care, paternity confidence and mating behaviour in mouthbrooding fishes.

## 1. Introduction

Paternal mouthbrooding is a parental care strategy where the male parent incubates eggs/larvae in the buccal cavity to protect the offspring from biotic and abiotic stressors [1,2]. Mouthbrooding involves a considerable investment from the adult, as the brooder is typically unable to feed, or at least is severely constrained [2,3]. Due to the high level of male investment [4], paternal mouthbrooding is generally assumed to be associated with high confidence in paternity (monoandry) [5]. The only two genetic parentage studies on paternal mouthbrooders, *Osteoglossum bicirrhosum* and *Sphaeramia nematoptera*, reported monogamous broods with the brooder male as the sire (73–92%), but revealed occurrences of multiple paternity (7.6–21%) for both species, multiple maternity (10.6%) for *S. nematoptera* and broods sired by a different male (18%) for *O. bicirrhosum* [6,7]. Investigation of genetic mating patterns is critical for illuminating individual reproductive strategies and behaviours, which may impact the species' reproductive success [8,9]. Because empirical data on the genetic parentage patterns of broods of paternal mouthbrooders are still poorly known, our understanding of the evolutionary drivers of this parental care strategy remains limited.

Male care, the predominant parental care type in fish, is theorized to be correlated with external fertilization, pair-spawning and paternity confidence [10–12]. Fish mating systems are also associated with the type of parental care exhibited [5]. Male care has been shown to correlate with high incidences of multiple maternity but low rates of multiple paternity, while in female and biparental care, this relationship is generally reversed [5]. Mixed parentages in broods can be a consequence of alternative reproductive behaviours within species such as cuckoldry in the male nesting *Lepomis punctatus* and fry adoption in the maternal mouthbrooding *Simochromis pleurospilus* [8,9,13–15]. For paternal mouthbrooders, broods may be sired by more than one male, by a different male or may consist of eggs from more than one female if some of the eggs received from the first female are partially cannibalized [6,7]. Because all the offspring and the putative father can be genetically sampled [5,6,9], genetic studies on paternal mouthbrooding can test hypotheses on male parental care including sexual selection, mating patterns and paternity confidence.

We investigate patterns of paternity in broods of two freshwater paternal mouthbrooders from divergent fish families, mouth almighty *Glossamia aprion* (Apogonidae) and blue catfish *Neoarius graeffei*. Although these species are fairly poorly studied, *G. aprion* spawns all year round, incubates for two to three weeks and has a fecundity of 104–532 and brood size of 4–416 eggs [16–21], Contrastingly, *N. graeffei* spawns during the wet season only, incubates for four to five weeks and has a fecundity of 4–128 and brood size of 1–88 eggs [17,18,21]. For both species, mouthbrooding comes at the expense of the parent's feeding, body condition and digestive and respiratory capacities [17]. While *G. aprion* has been observed to form mating pairs in aquaria [22], the detailed reproductive activity (e.g. fertilization step) of both species has never been observed [18]. Based on previous studies [5–7], we predict that the broods of both species are genetically related to the brooder male; support for this prediction will provide justification for paternity confidence in these two study species and for their paternal mouthbrooding strategy. We also predict that the broods of paternal mouthbrooders will show higher rates of multiple maternity relative to multiple paternity.

## 2. Materials and methods

### (a) Sample collection and preparation

Samples of 115 *N. graeffei* (nine brooding males, 90 offspring and 16 non-brooding adults) and 261 *G. aprion* (18 brooding males, 180 offspring and 63 non-brooding adults) were collected across rivers of Northern Territory, Australia. *G. aprion* was collected in five sites: the Mary River (AHMR), Adelaide River (DRAR) and Daly River (OODR, SHFR, GJKR) and *N. graeffei* was collected from one site (Mary River) using electrofishing. Non-brooding males and females were included to provide the basic population genetics context for our study including allele frequency estimates required for parentage analysis. Each fish was euthanized in AQUI-S (175 mg l^−1^ for 20 min) upon capture; brooding males together with their offspring were bagged individually, labelled and kept in an ice slurry until processed (see electronic supplementary material, S1–S3 for fish collection and justification of method). Fish collections were made as part of a larger study investigating trait variation in freshwater fishes, thereby using specimens for multiple research purposes. These species are abundant, widely distributed in the northern Australian rivers and not considered threatened species [23].

We randomly sampled 9–11 offspring from each brooding male parent. This sample size gave us a 95% chance of detecting the contribution of more than one parent (of either sex) to a brood as long as the dominant parent contributed at least 22–30% of the offspring ([5,8]; see electronic supplementary material, S4 for explanation). Approximately 5–7 mg of adult muscle, larval tissue or whole egg was collected and stored in 70% ethanol until DNA extraction and genomic sequencing.

### (b) Single-nucleotide polymorphism discovery and filtering

Samples were genotyped at single-nucleotide polymorphism (SNP) loci using the DArTSeq method at Diversity Arrays Technology Pty Ltd, Canberra, Australia [24]. We used strict SNP filtering criteria to retain only high confidence genotypes for parentage analysis using a combination of custom R scripts, ‘adegenet’, ‘dartR’ and ‘plotly’ packages in R [25–28] (see electronic supplementary material, S5 for detailed SNPs filtering and genetic diversity estimation methods).

### (c) Parentage and sib-ship inference analysis

We first examined the percentage of opposite homozygotes (%OH) between each offspring in the brood and their brooding parent to help identify typing error rates and possible non-paternity of brooding male using the CalcOHLLR function in the ‘sequoia’ R package [27,29]. We also examined the %OH between non-parents and each offspring in a brood by randomly calculating the OH of each offspring with another brooding male. To determine whether the brooding male parent is the true father of each brood and whether each brood consisted of full- or half-siblings, we performed a full-likelihood parentage analysis using the Colony(v. 2) software [30]. Colony was run using moderate prior, medium run, assumed polygamy for both males and females, and using a range of error rates (0.01–1%) informed by %OH calculations.

## 3. Results

### (a) Single-nucleotide polymorphism discovery and filtering

From 23 963 SNPs, 1748 SNPs (nine brooding males, 90 offspring and 13 non-brooding adults) remained for analysis in *N. graeffei* after filtering with a minimum and mean sequencing depth of 20 and 30.98 per genotype, respectively. From 12 351 SNPs, 1313 SNPs (15 brooding males, 146 offspring and 58 non-brooding adults) remained for analysis in *G. aprion* after filtering with a minimum and mean sequencing depth of 20 and 64.62 per genotype, respectively. See table 1 for SNPs metrics of *N. graeffei* and *G. aprion*. For both species, SNP PIC scores were greater than 0.20 and had 1.19% (*N. graeffei*) and 1.26% (*G. aprion*) missing data. (See electronic supplementary material, S6–S9 for genetic diversity indices of the sampled population.)

**Table 1.** SNPs metrics (average ± s.d.) of *Neoarius graeffei* and *Glossamia aprion* before and after SNP filtering.

	*n* loci	call rate	mean sequencing depth	mean allele depth ratio	repeatability
*Neoarius graeffei*					
SNPs pre-filtering ± s.d. (all samples)	23963	0.82 ± 0.22	16.99 ± 10.05	1.51 ± 0.52	0.99 ± 0.02
SNPs post-filtering ± s.d. (all samples)	1748	0.99 ± 0.02	30.98 ± 8.24	1.46 ± 0.27	1
SNPs post-filtering ± s.d. (adults only)	1748	0.99 ± 0.02	30.98 ± 8.24	1.46 ± 0.27	1
*Glossamia aprion*					
SNPs pre-filtering ± s.d. (all samples)	12 351	0.85 ± 0.18	37.78 ± 41.6	1.52 ± 0.6	0.98 ± 0.02
SNPs post-filtering ± s.d. (all samples)	1313	0.98 ± 0.02	64.62 ± 40.31	1.30 ± 0.24	1
SNPs post-filtering ± s.d. (adults only)	1313	0.98 ± 0.02	64.63 ± 40.31	1.29 ± 0.24	1

### (b) Percentage of opposite homozygotes, genetic parentage and sib-ship inference

**Figure 1.**
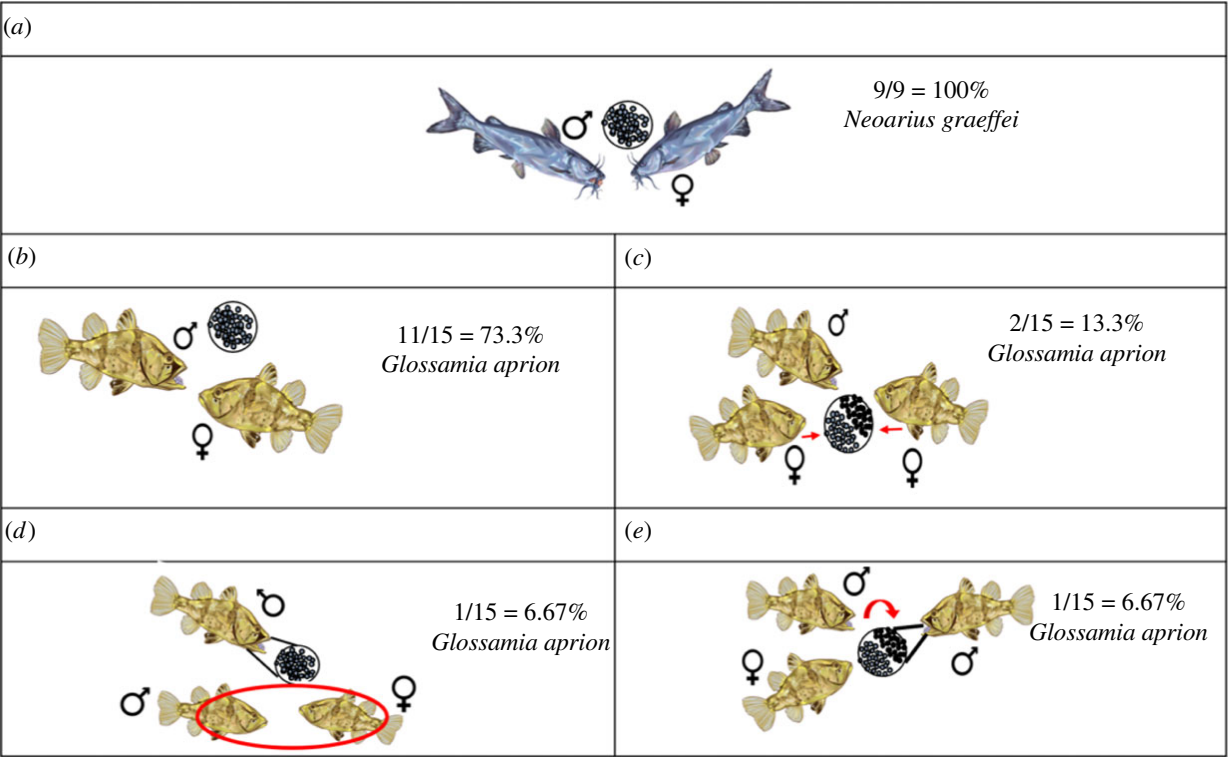
Parentage patterns of *Neoarius graeffei* and *Glossamia aprion* showing (a,b) monogamous brood with the brooder male as the sire: (*a*) one mother and one father in a brood and (*b*) one mother and one father in a brood; (*c*) brood with more than one mother (two mothers) and one father in a brood; (*d*) brood genetically unrelated to the brooder male; and (*e*) brood with more than one father (two fathers) and one mother in a brood.

For *N. graeffei*, the offspring in each brood were assigned as full siblings with the brooder male as the sire. The %OH between offspring-brooding parent pair ranged from 0.81 to 1.98%, where we assume that the brooding male is the parent. This range gives an estimate of the genotyping error rate between the brooding parent and its offspring, assuming that the lower bound is closer to the true error rate and the upper bound due to non-paternity of some offspring. By contrast, the %OH of known non-parents and each offspring range from 6.1 to 10%, which represents the estimated %OH of non-paternity. At 1% marker error rate, parentage assignment showed that all broods (*n* = 9/9) were assigned to their brooding male parent. All the broods were also assigned as full siblings with an inclusion and exclusion probability range of 0.999–1.00 (figure 1*a*; electronic supplementary material, S10 and S11)*.* At 0.01% error rate, *N. graeffei* broods were assigned as full siblings but the brooding male was excluded as the sire. We assumed that this is due to genotyping error rate given that the %OH is much lower than that for known non-parents.

For *G. aprion*, the parentage patterns were more heterogeneous between the investigated broods. The %OH between each offspring and its respective brooding parent examined ranged from 0 to 4.3%. Between known non-parents and each offspring*,* the %OH ranges from 0.15% to 37.75%. Parentage assignment showed that the *G. aprion* broods exhibited heterogeneous mating patterns with an inclusion and exclusion probability range of 0.081–1.00 (figure 1*b*–*e*; electronic supplementary material, S10 and S11). At 1% and 0.01% error rates, 11 of the 15 broods (73%) were assigned as full siblings with the brooder male as the sire (figure 1*b*). Two broods (13%) had two females contributing to the broods' genotype (figure 1*c*), one brood (6%, GA355) was genetically unrelated to the brooder male (figure 1*d*), and another brood (6%) was fathered by more than one male (figure 1*e*).

## 4. Discussion

While the certainty of paternity is theorized to co-evolve with male care [10–12], there are many reports of male care in fishes with incidences of multiple paternity and alternative reproductive behaviours [5,8,9,13–15]. Unlike other types of male care in fishes, there is still little genetic information available on broods of paternal mouthbrooders [5–7]. In this study, we revealed a largely monogamous genetic mating pattern for the two study species. *N. graeffei* showed monogamous broods with the brooder male as the sire, supporting our prediction that the mouthbrooding male was the true father of the offspring. *G. aprion* broods, however, revealed polygyny, adoption and cuckoldry in a background of a mostly monogamous mating pattern. These results suggest intraspecific variability in the genetic mating pattern of *G. aprion*.

Parental care in mouthbrooders affords a very high cost to the care-giving male [17,31,32], so the overall benefit of care depends on balancing fitness cost with a high certainty in parentage [11,33]. The broods of both species were largely monogamous which is likely associated with the relative costs and benefits of their paternal mouthbrooding strategy. There are evolutionary incentives for male carers to provide care if they are the only male progenitor of their brood because the fitness returns of caring trades-off against the energy invested [34–36]. The energetic costs associated with paternal mouthbrooding may limit the brooder's ability to mate with more than one female [4,17,31,32]. Moreover, the limited capacity of the buccal cavity to accommodate eggs from another female may also restrict male parents from engaging in extra-pair mating [37,38].

The contrasting reproductive ecologies and level of paternal investment may have contributed to the variations in the parentage patterns observed in the two study species. *N. graeffei* broods fewer eggs (1–88) and for a longer duration (four to five weeks) than *G. aprion* which broods 4–512 eggs for two to three weeks [17,18]. Spawning for *N. graeffei* occurs in the highly interconnected and productive wet season, while *G. aprion* spawns throughout the year, with higher occurrence during the wet–dry seasons, where young are exposed to habitat restriction and concentration of organisms (predators, competition) during the extended dry season [21]. In this study, *G. aprion* had considerable variation in genetic mating behaviour. Variation in mating behaviour within species is suggested to increase the success of passing on multiple gene types in response to varying ecological factors in the environment [39], which seems a plausible justification for *G. aprion*.

The two *G. aprion* broods, (i) fathered by more than one male and (ii) genetically unrelated to the brooder male, could possibly be explained by cuckoldry and adoption of brood, respectively [6–9]. From observations in aquaria, the male and female pair off during courtship; the female then releases the egg mass, and the male begins taking it in its buccal cavity when it is half-way out of the female [22]. A female was also seen to chase the brooding male making it spit its eggs—which the male did not take back [22]. Such behavioural accounts in *G. aprion* likely provide an opportunity for other non-brooding males to take another's brood and care for them, and also for the male to ‘swap’ broods. While there is apparently little genetic benefit for males to care for the offspring of another, females may preferentially choose males who are already caring for progeny [40].

In broods with multiple maternity, *G. aprion* males could have mated with a female with only a few eggs or cannibalized a portion of their brood when presented with a new mating opportunity. This is likely since the fecundity of *G. aprion* females were reported to range from 104 to 532 eggs [17,18]. As seen in aquaria, females may also approach brooding males and try to mate with them [22]. Brooding *S. nematoptera* males, a confamilial of *G. aprion*, were reported to receive a new egg clutch from another female in the wild [7]. Multiple mating by males may be a consequence of females being attracted to males that are already mouthbrooding [40] or a strategy to replenish the brooder's energy reserves to maintain condition while brooding [41].

We uncovered novel insights into the poorly known mating behaviour of paternal mouthbrooding fishes in the wild. While *N. graeffei* and *G. aprion* broods were largely monogamous with the brooder male as the sire, *G. aprion* surprisingly exhibited some degree of heterogeneity in its mating pattern—a potential reflection of alternative reproductive behaviours that aim to increase reproductive success. We highlight the potential for complex interrelationships of male care, paternity confidence and mating behaviour in mouthbrooding fishes, and suggest caution in generalized assumptions of parentage of animals displaying parental care.

## Data Availability

Data and codes are available in the Dryad Digital Repository: https://doi.org/10.5061/dryad.905qfttmn [42]. The data are provided in the electronic supplementary material [43].
